# Supplementary material for: Transcriptomic response in symptomless roots of clubroot infected kohlrabi (Brassica oleracea var. gongylodes) mirrors resistant plants
Source: BMC Plant Biol. 2019 Jul 1;19:288. doi: 10.1186/s12870-019-1902-z (PMC6604361; doi:10.1186/s12870-019-1902-z)
Supplement: Supplementary file 1 — Figure S1. Kohlrabi (Brassica oleracea) and Plasmodiophora brassicae reads. Samples from the symptomless roots (SL) of infected plants contained less than 0.0005% P. brassicae reads. P. brassicae read partition increased from 23% in WG to 33% in BG. Figure S2: Kohlrabi flavonoid metabolism. Clustered heatmaps of log2 fold change values of DEGs. No DEGs were present comparing WG with BG. Arabidopsis homologs are given. NA: not assigned. Figure S3: Kohlrabi auxin response factors. Clustered heatmaps of log2 fold change values of DEGs. One DEG (AXR3) was present comparing WG with BG. Arabidopsis homologs are given. Figure S4: Kohlrabi myrosinases and nitrilases. Clustered heatmaps of log2 fold change values of DEGs. No DEGs were present comparing WG with BG. Arabidopsis homologs are given. NA: not assigned. Figure S5: Kohlrabi WRKY and bZIP transcription factors. Clustered heatmaps of log2 fold change values of DEGs. No DEGs were present comparing WG with BG. Arabidopsis homologs are given. NA: not assigned. Figure S6: KEGG map for plant-pathogen interaction. Up-regulated genes in SL compared with galls are shaded in purple and down-regulated genes in green. Genes shaded in purple and green were up- and down-regulated in different homologs or isoforms. Figure S7: Kohlrabi kinases. Clustered heatmaps of log2 fold change values of DEGs. No DEGs were present comparing WG with BG. Arabidopsis homologs are given. NA: not assigned. Figure S8: Numbers of P. brassicae genes in clubroot infected kohlrabi roots per COG category. Bars indicate total genes found across all libraries. Unassigned genes (n = 5482) are not illustrated. Figure S9: Cumulated FPKM values of P. brassicae reads obtained from WG and BG samples. Unassigned genes are not illustrated. Figure S10: Cumulated FPKM values of P. brassicae reads obtained from SL samples. Unassigned genes are not illustrated. (DOCX 2519 kb) [file 12870_2019_1902_MOESM1_ESM.docx]

**Transcriptomic response in symptomless roots of clubroot infected kohlrabi mirrors resistant plants**

**Supplementary Figures**

Stefan Ciaghi^1,$^, Arne Schwelm^1,2,$^, Sigrid Neuhauser^1,*^

*1 University of Innsbruck, Institute of Microbiology, Technikerstraße 25, 6020 Innsbruck, Austria*

*^2^ Swedish University of Agricultural Sciences, Department of Plant Biology, Uppsala BioCenter, Linnean Centre for Plant Biology, P.O. Box 7080, SE-75007 Uppsala, Sweden*

^*^ Correspondence: Sigrid.Neuhauser@uibk.ac.at

^$^ Contributed equally to this work


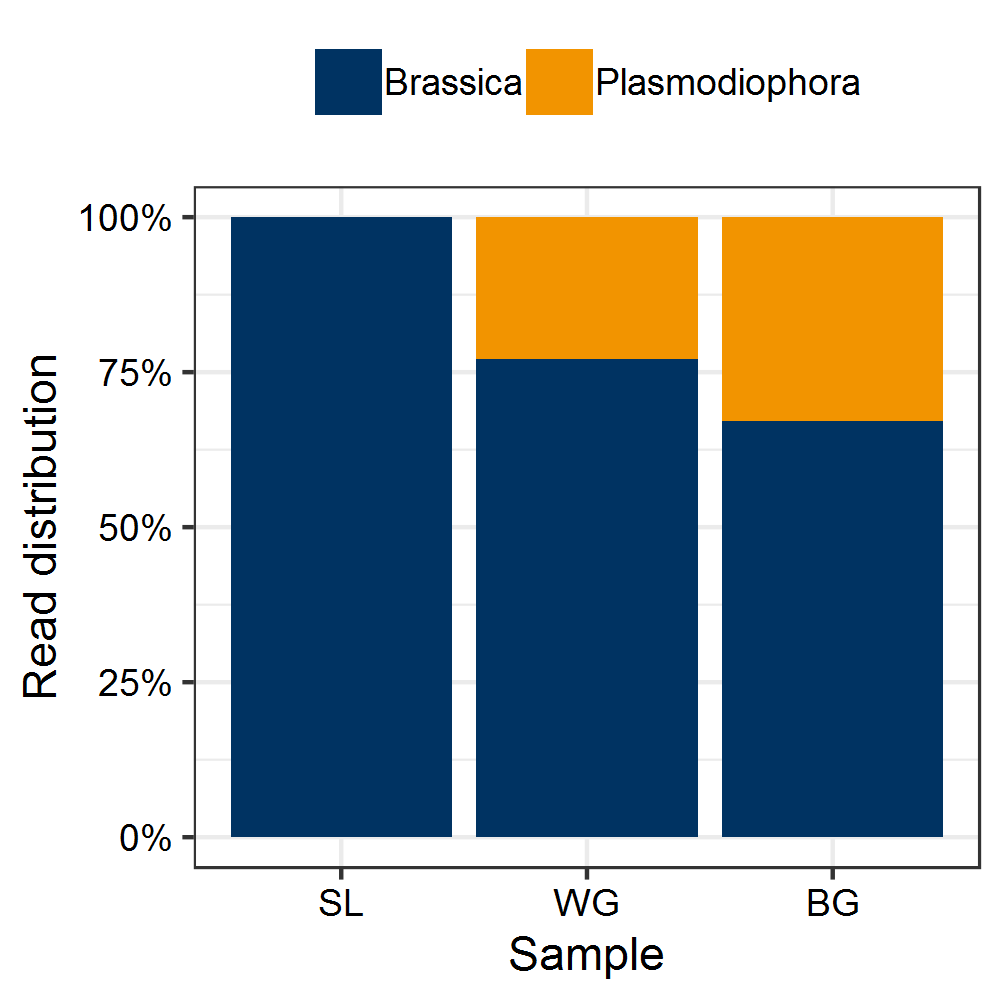


**Figure S1:** **Kohlrabi (*Brassica oleracea*) and *Plasmodiophora brassicae* reads.** Samples from the symptomless roots (SL) of infected plants contained less than 0.0005% *P. brassicae* reads. *P. brassicae* read partition increased from 23% in WG to 33% in BG.


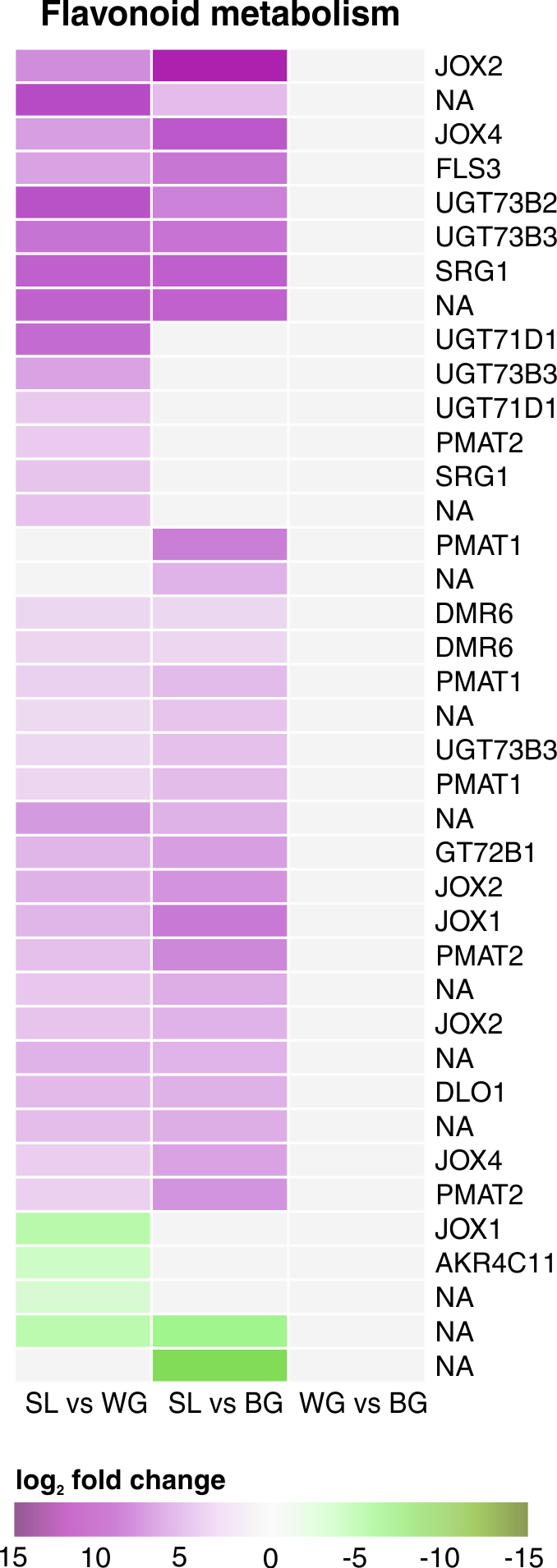


**Figure S2:** **Kohlrabi flavonoid metabolism.** Clustered heatmaps of log_2_ fold change values of DEGs. No DEGs were present comparing WG with BG. Up-regulated genes are shaded in purple and down-regulated genes in green. *Arabidopsis* homologs are given. NA: not assigned.


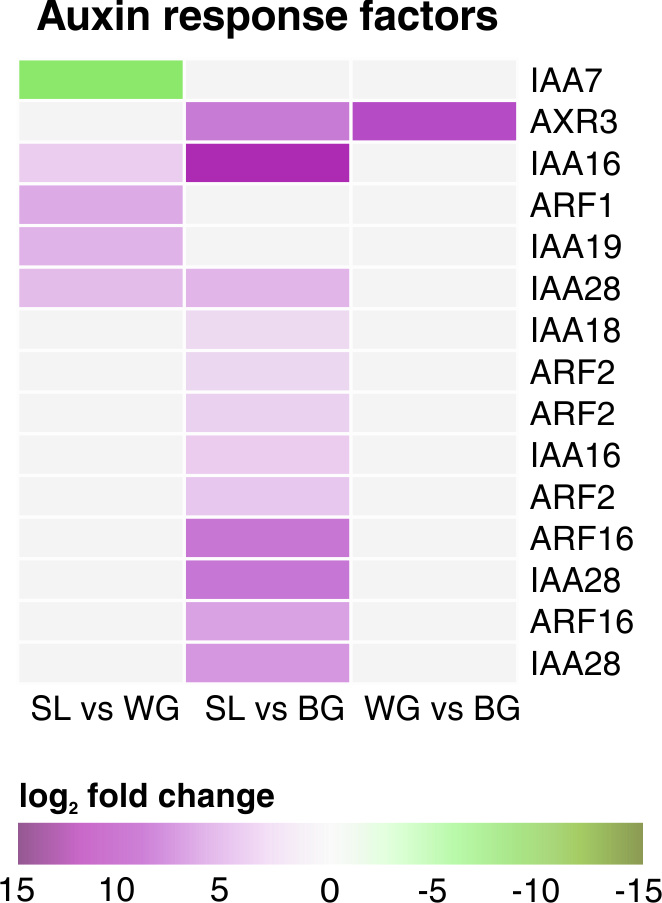


**Figure S3: Kohlrabi auxin response factors.** Clustered heatmaps of log_2_ fold change values of DEGs. One DEG (AXR3) was present comparing WG with BG. Up-regulated genes are shaded in purple and down-regulated genes in green. *Arabidopsis* homologs are given.


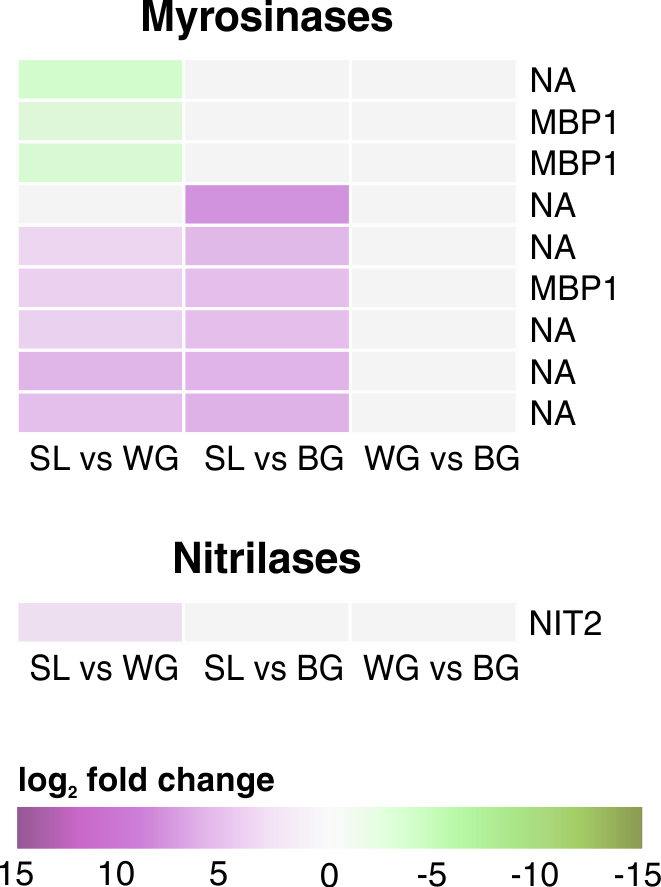


**Figure S4: Kohlrabi myrosinases and nitrilases.** Clustered heatmaps of log_2_ fold change values of DEGs. No DEGs were present comparing WG with BG. Up-regulated genes are shaded in purple and down-regulated genes in green. *Arabidopsis* homologs are given. NA: not assigned.


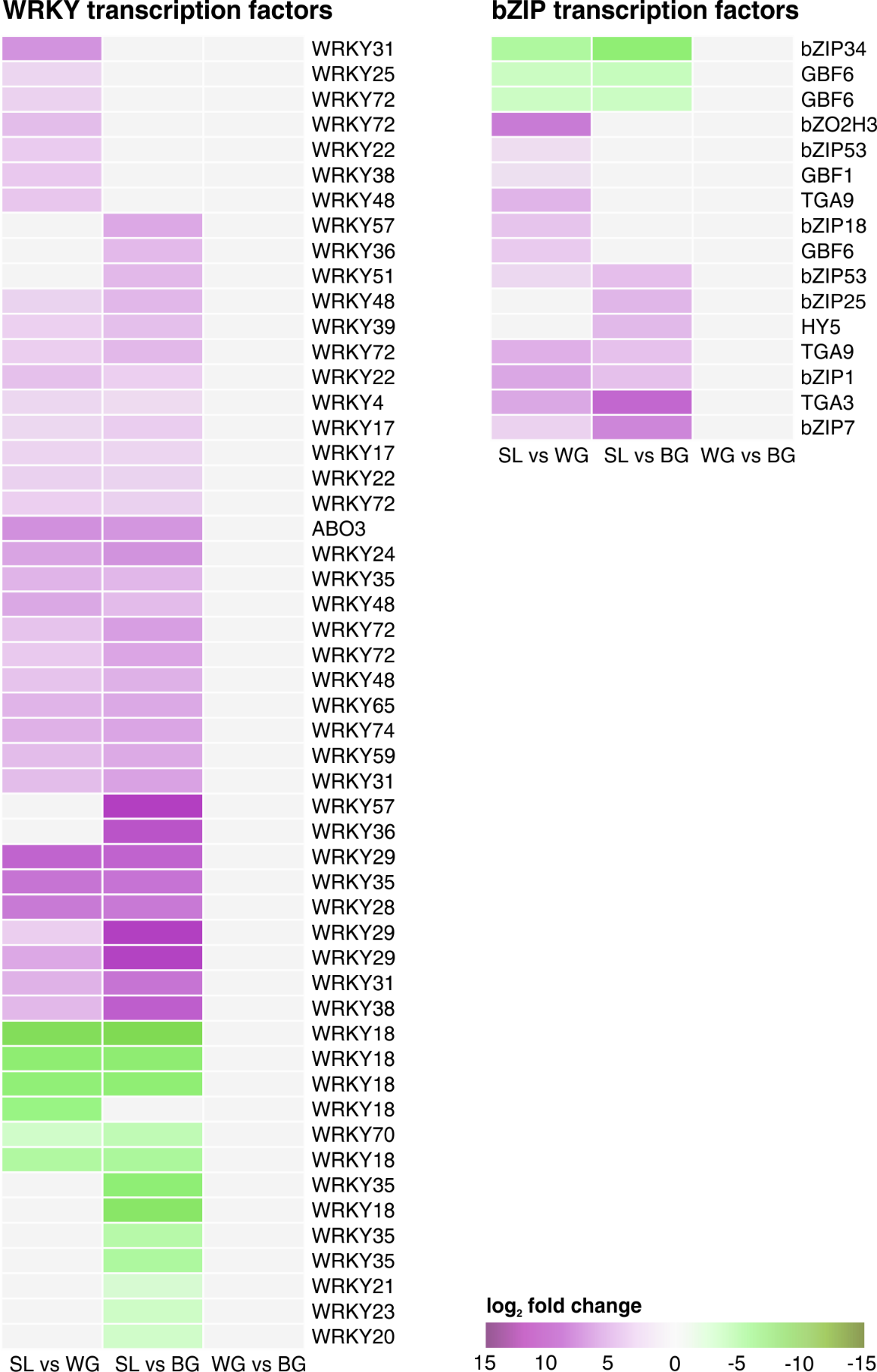


**Figure S5: Kohlrabi WRKY and bZIP transcription factors.** Clustered heatmaps of log_2_ fold change values of DEGs. No DEGs were present comparing WG with BG. Up-regulated genes are shaded in purple and down-regulated genes in green. *Arabidopsis* homologs are given. NA: not assigned.


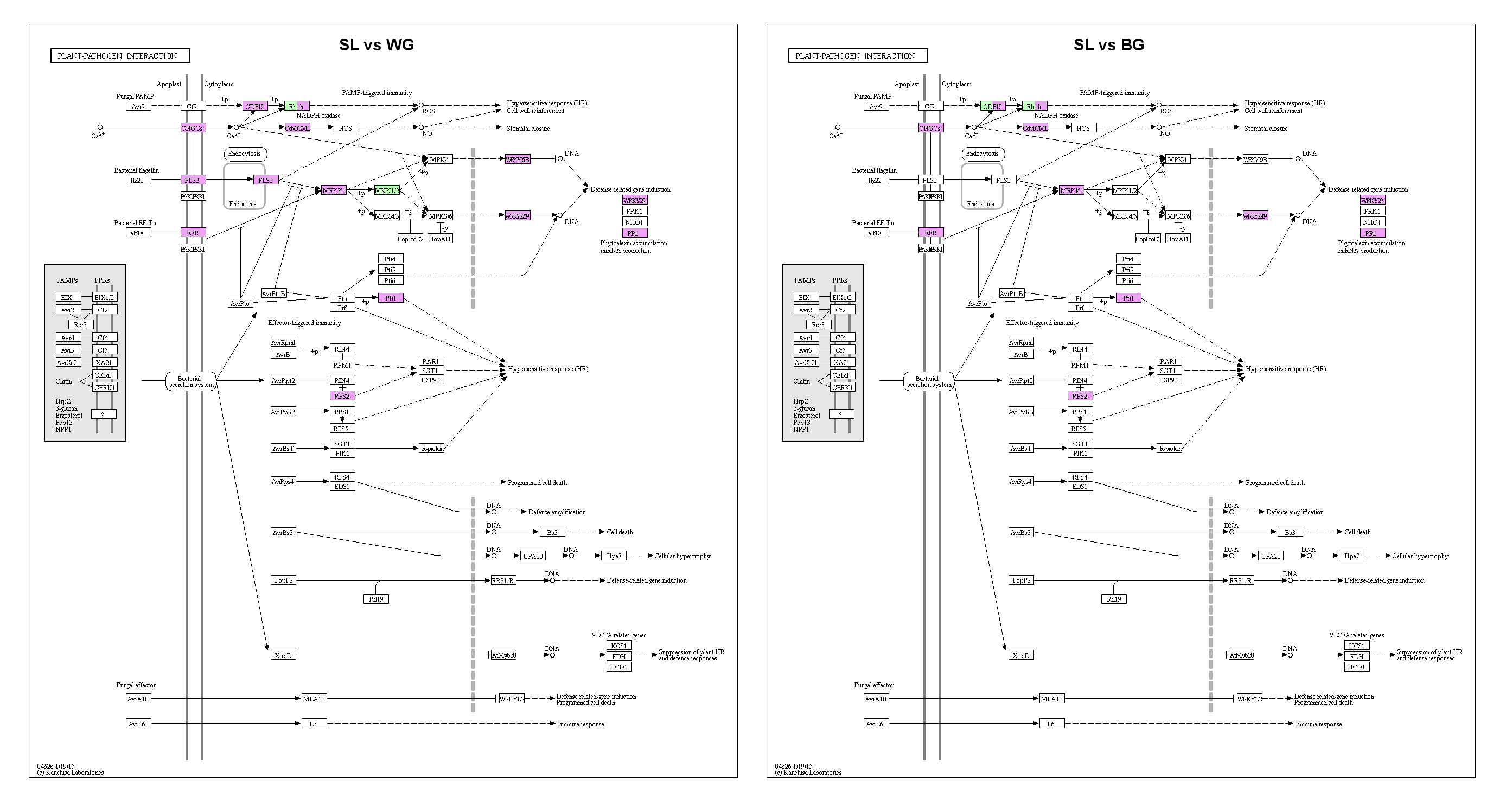


**Figure S6: KEGG map for plant-pathogen interaction.** Up-regulated genes in SL compared with galls are shaded in purple and down-regulated genes in green. Genes shaded in purple and green were up- and down-regulated in different homologs or isoforms.


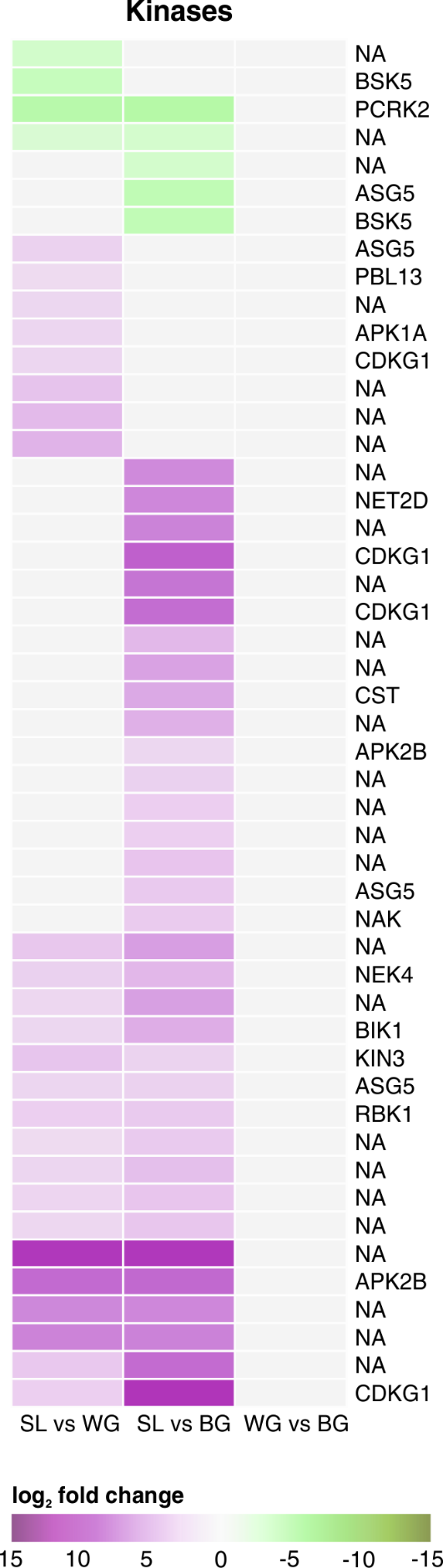


**Figure S7: Kohlrabi kinases.** Clustered heatmaps of log_2_ fold change values of DEGs. No DEGs were present comparing WG with BG. Up-regulated genes are shaded in purple and down-regulated genes in green. *Arabidopsis* homologs are given. NA: not assigned.


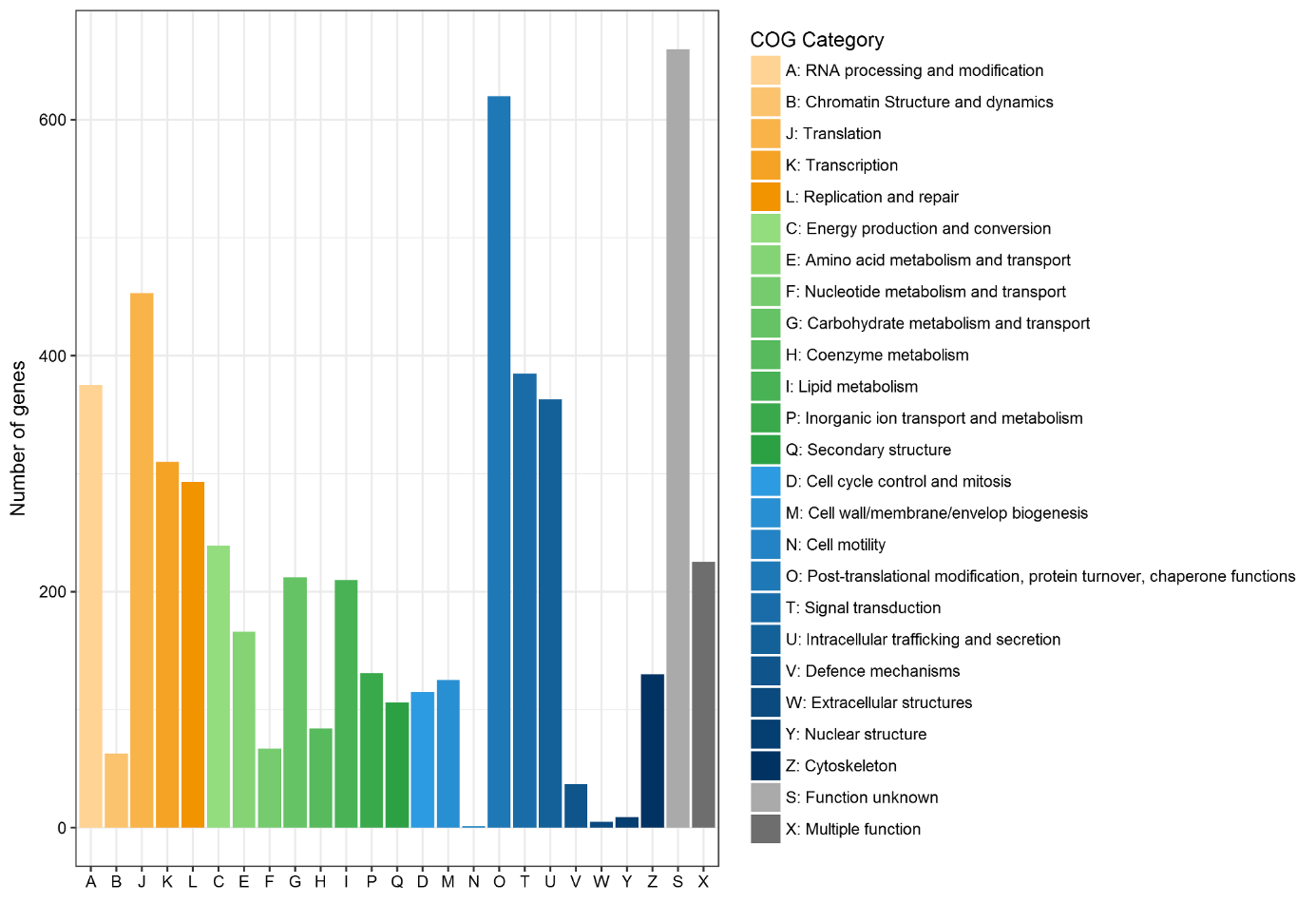


**Figure S8:** **Numbers of *P. brassicae* genes in clubroot infected kohlrabi roots per COG category.** Bars indicate total genes found across all libraries. Unassigned genes (n = 5482) are not illustrated. Orange: Information and Storage Processing; Green: Metabolism; Blue: Cellular Process and Signalling; Grey: Poorly Characterized.


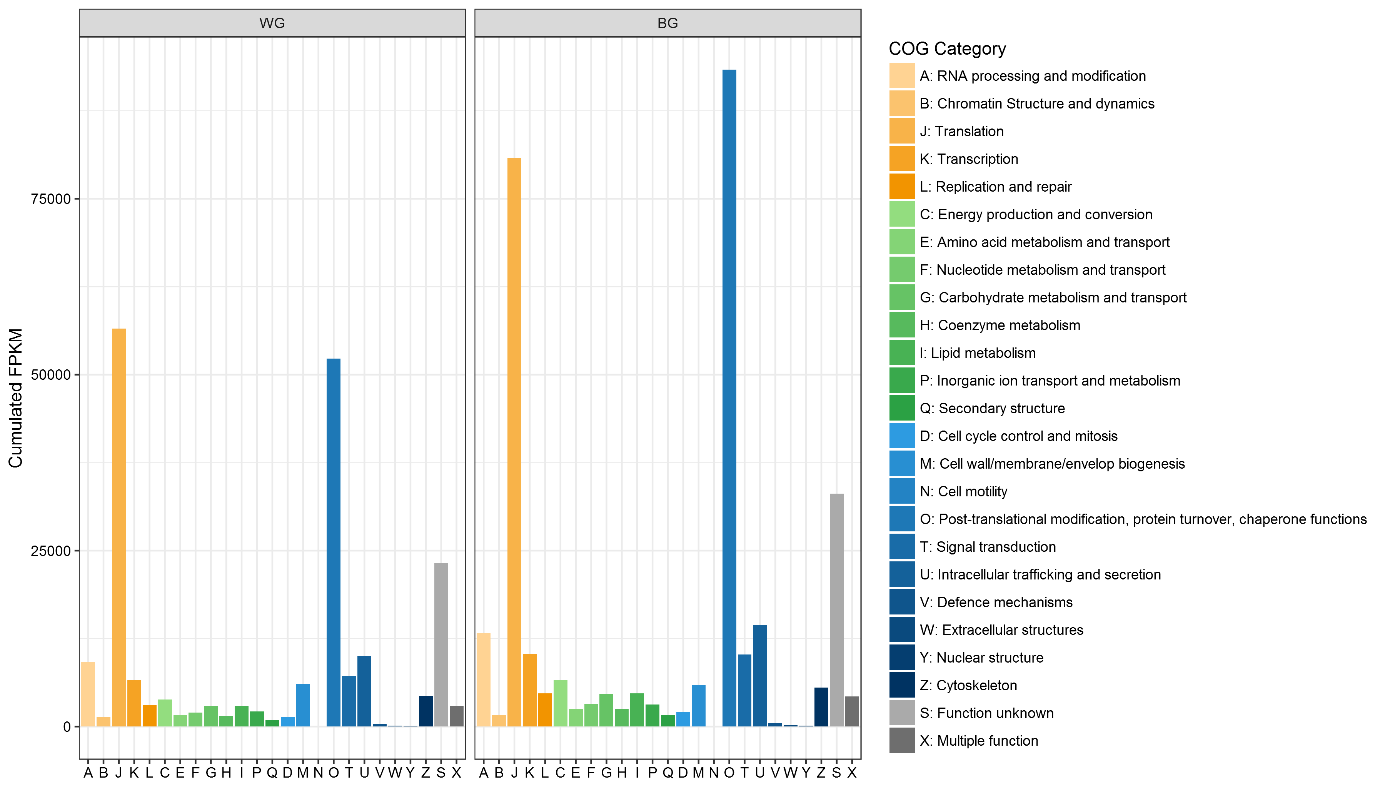


**Figure S9:** **Cumulated FPKM values of *P. brassicae* reads obtained from WG and BG samples.** Unassigned genes are not illustrated. Orange: Information and Storage Processing; Green: Metabolism; Blue: Cellular Process and Signalling; Grey: Poorly Characterized.


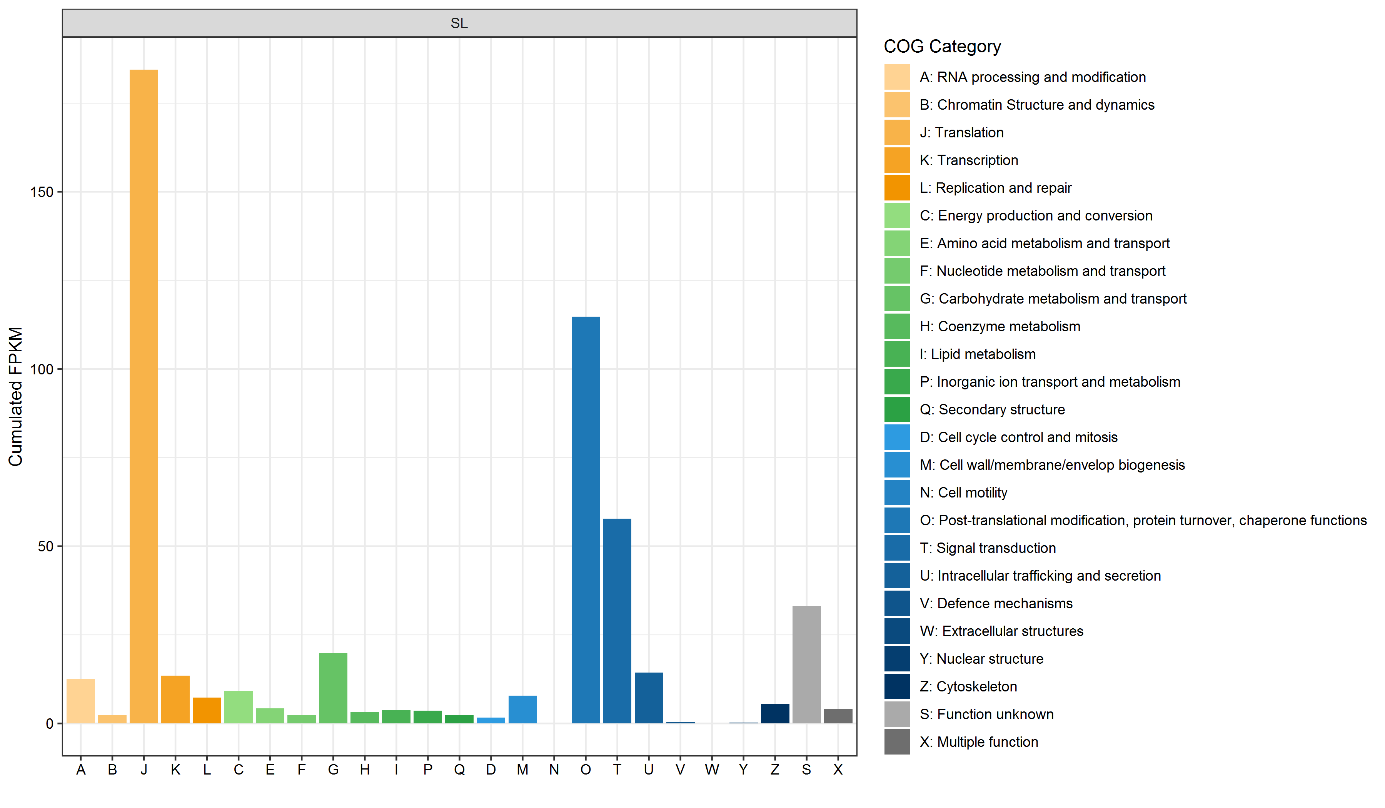


**Figure S10:** **Cumulated FPKM values of *P. brassicae* reads obtained from SL samples.** Unassigned genes are not illustrated. Orange: Information and Storage Processing; Green: Metabolism; Blue: Cellular Process and Signalling; Grey: Poorly Characterized.
